# Impact of soft-tissue management techniques on immediate implant placement: a randomized controlled trial

**DOI:** 10.1186/s40729-026-00667-5

**Published:** 2026-01-27

**Authors:** Frederic Bouffleur, Andreas Ruoss, Reinald Kühle, Christopher Büsch, Michael Engel, Jürgen Hoffmann, Christian Mertens

**Affiliations:** 1 Department of Oral- and Cranio‐Maxillofacial Surgery, Heidelberg University Hospital, Im Neuenheimer Feld 400, 69120 Heidelberg, Germany; 2 Institute of Medical Biometry, University Heidelberg, Im Neuenheimer Feld 130.3, 69120 Heidelberg, Germany

**Keywords:** Immediate implant placement, Prospective randomized clinical study, Implant

## Abstract

**Purpose:**

In immediate implant placement, both open and closed healing techniques are used, but their comparative esthetic and tissue stability outcomes remain under debate. This study aimed to evaluate and compare these two approaches to support clinical decision-making.

**Methods:**

In this prospective, randomized controlled trial, 46 patients received a total of 48 implants, assigned to either an open healing group (*n* = 25) or a closed healing group (*n* = 23). Clinical and radiological assessments were conducted at three time points: T1 (pre-extraction), T2 (3 months post-op), and T3 (12 months post-op). The primary endpoint was the Pink Esthetic Score (PES), which assesses seven soft tissue parameters. Secondary endpoints included implant survival and volumetric tissue changes. PES was analyzed using a linear mixed-effects model.

**Results:**

The open healing group showed significantly higher PES outcomes compared to the closed healing group (mean difference: -1.49; 95% CI: [-2.36, -0.62]; *p* = 0.0014). A higher baseline PES was also significantly associated with better esthetic outcomes at follow-up (0.3638, 95% CI: [0.1890, 0.5386], *p* = 0.0002). Smoking had no significant effect. Volumetric analysis revealed soft tissue volume loss from T1 to T2, with partial recovery by T3. Although the open healing group showed slightly less volume loss, this was not statistically significant. No implant losses occurred in either group.

**Conclusion:**

The study demonstrated esthetic advantages of the open healing technique compared to the closed healing technique in immediate implant placement.

## Introduction

 Immediate implant placement (IIP) has become one of the most compelling treatment modalities in the replacement of missing dentition, offering the dual advantages of shorter treatment time and preservation of the alveolar architecture [1]. Critical to the success of IIP is the achievement of adequate primary stability, which is not only a prerequisite for the immediate loading protocol but also the basis for subsequent therapeutic steps. Optimal primary stability allows immediate provisionalization and is associated with better hard and soft tissue results, especially in the esthetically demanding anterior region [2–5].

In cases where primary stability is not sufficient or, as a surrogate parameter, the insertion torque is insufficient for immediate loading, two different strategies are generally possible to enable successful osseointegration and peri-implant tissue healing. The first strategy is covered healing, in which a cover screw is inserted after flap elevation and soft tissue mobilization. This technique creates a closed environment that protects both the implant and the simultaneously placed graft material during the early stages of osseointegration. However, elevation of the flap requires additional surgical trauma, which can result in coronal displacement of the keratinized mucosa towards the alveolar base and contribute to underlying bone resorption, which has been reported to be up to 0.5 mm [4, 6, 7]. This resorption is particularly critical in the anterior zone, where the buccal bone is often less than 0.5 mm thick [8], and can compromise soft tissue support and esthetic outcome due to increased implant visibility and exposure.

Alternatively, an open healing approach employs the use of a healing abutment in conjunction with adaptive suturing, thereby eliminating the need for flap elevation and subsequent second-stage surgery. This method is less invasive and may enhance patient comfort; however, the exposure of the implant and grafting materials to the oral environment can adversely affect soft tissue healing and esthetics, especially in the anterior region, by increasing the loss of augmented volume and recession of keratinized gingiva [9].

Given these considerations, the present randomized controlled clinical trial aimed to compare closed healing with a cover screw versus open healing with a healing abutment in the context of IIP. Specifically, we evaluated the impact of these techniques on the esthetic outcome and stability of hard and soft tissues.

## Materials and methods

This explorative study was conducted as a randomized controlled clinical trial, approved by the Ethics Committee of the Medical Faculty of the University of Heidelberg (application S-230/2017), and in accordance with the current principles of the World Medical Association’s Declaration of Helsinki. Prior to enrolment, all patients provided signed informed consent. The data are reported in accordance with the Consolidated Standards of Reporting Trials (CONSORT [10]) statement for standardized reporting of randomized clinical trials. Patients were enrolled from July 2017–November 2020 in two parallel groups in a 1:1 ratio by an experienced oral surgeon in a dental practice specializing in dental implants. No changes to the study protocol were made after the start of the enrollment. The trial was retrospectively registered (DRKS00038390).

### Inclusion criteria

Patients were eligible for inclusion if they required single-tooth extraction in the anterior or premolar tooth position (FDI (Fédération dentaire internationale) position 015–025 and 035–045) and requested a single implant-retained restoration. Multiple implantations in one patient were possible if all inclusion and exclusion criteria were fulfilled.

- IIP, directly after tooth extraction.
- Sufficient bone volume to allow IIP.
- Adjacent teeth present for reference.

Exclusion criteria.

Patients were not included in the study if one or more exclusion criteria were present:

- Systemic disease (e.g., diabetes mellitus).
- Previous irradiation treatment.
- A history of intravenous bisphosphonate therapy.
- Smokers of > 10 cigarettes/day.
- Untreated periodontitis or dental caries.
- Patients under 18 years of age.
- Poor oral hygiene or compliance.
- Missing adjacent teeth.
- Insufficient apical bone volume (min. 3 mm).
- Large dehiscences or fenestrations of buccal bone.
- Extensive inflammation.
- Compromised alveolar buccal wall.
- Immediate loading and immediate provisionalization.
- < 30 Ncm insertion torque.

## Preoperative analysis

### Soft-tissue analysis

To assess preoperative soft tissue conditions and analyze subsequent changes in the gingiva and surrounding structures, impressions were systematically obtained at various time intervals. The initial impression, created on day 0, was established as the baseline (T1). Subsequent impressions were acquired at specific intervals, namely three months (T2), and 12 months (T3).

All impressions were consistently executed by the same clinician, ensuring methodological uniformity. All impressions were poured with Type IV dental stone. Conventional impressions were selected over intraoral scanning due to their superior accuracy in capturing soft tissue contours, particularly in edentulous areas. While intraoral scanners are widely accepted for imaging teeth and keratinized gingiva, conventional impressions followed by model digitization have been shown to provide more reliable data for the assessment of peri-implant soft tissues and mucosal architecture. Subsequently, the resulting models were subjected to digitization using a 3D laboratory scanner (map400, Ceramill, Pforzheim, Germany, Accuracy: 0,17 ± 0,01 mm). The resulting digital files in Standard Triangle Language (STL) format were harmonized through the utilization of dedicated software (ExoCad, Darmstadt, Germany), employing anatomical landmarks for precise alignment. To ensure accurate superimposition across the three time points (T1, T2, T3), a two-step alignment protocol was used: initial three-point alignment based on stable anatomical landmarks (e.g., palatal rugae or non-remodeled teeth), followed by a global refinement using Iterative Closest Point (ICP) registration. RMSE values showed fitting errors below 0.1 mm, which is considered acceptable for high-precision soft tissue analysis in implant dentistry. The workflow used is an established algorithm previously employed in other studies with excellent intra-operator reliability as previously reported [11].

### Radiographic analysis

A cone beam computed tomography (CBCT) scan was performed as part of preoperative diagnostics before tooth extraction and implant placement (T1). The data was used to measure bone dimensions and the integrity of the buccal bone. At the 12-month follow-up appointment (T3) a further CBCT scan was performed. To evaluate alveolar dimensions, cone beam computed tomography (CBCT) images were analyzed using dedicated imaging software (i-Dixel, J. Morita Corp, Japan). All measurements were performed by a single experienced examiner who was blinded to group allocation to reduce the risk of bias. Given that standardized and reproducible measurement protocols were applied within the software, intra- and inter-rater reliability were not assessed, as the measurements followed established procedures commonly used in implant-related radiographic evaluations.

To ensure anatomical consistency and minimize the influence of therapeutic interventions, the lowest point of the nasal floor was selected as a fixed anatomical reference in the upper jaw. In the lower jaw the inferior mandibular border was used. From this point, a horizontal line was drawn across the sinus to the vestibular aspect of the implant site. The vertical distance from this reference line to the highest point of the mesial bone was then measured at a 90° angle. The zoom function was also employed during this step to accurately define measurement points.

### Primary objective

In the anterior region, recession on implants is considered an esthetic failure, even if the implants have successfully osseointegrated. Therefore, it is crucial to maintain stable soft tissue after tooth extraction, particularly in the anterior maxilla.

The objective of this study was to establish a reproducible and esthetically satisfactory procedure for IIP. Two surgical techniques were compared based on their results in terms of soft tissue stability. Therefore, the Pink Esthetic Score (PES) was used as a well-established scoring method for gingival soft tissue esthetics [12]. The clinical parameters were evaluated during clinical examination by a single calibrated examiner who was not involved in the treatment of the patients, at T1 regarding the original tooth, at T2 after insertion of the restoration and at T3 12 months after implantation. The examiner had over four years of clinical experience in implant prosthodontics and esthetic soft tissue evaluation. Calibration was conducted prior to data collection using five representative example cases. These cases were independently scored by the examiner and then reviewed in consultation with a senior prosthodontist with more than 30 years of experience. Discrepancies were discussed until consensus was reached, ensuring the examiner’s understanding and consistent application of the PES criteria. This calibration process helped to standardize esthetic assessments and supported the reproducibility of outcome measurements.

### Primary endpoint

As primary endpoint the preoperative summary PES at baseline (T1) as well as postoperative PES at three months (T2) and 12 months (T3) was used. The evaluation was performed by comparing the replaced tooth to the contralateral tooth in direct clinical examination. The summary PES was defined as the sum of the following seven parameters:

- Mesial papilla.
- Distal papilla.
- Level of soft-tissue margin.
- Soft-tissue contour.
- Alveolar process deficiency.
- Soft-tissue color.
- Soft tissue texture.

A score of 2, 1, or 0 was assigned to all PES parameters (0 = lowest value, 2 = highest value, higher scores are better). Hence, the maximum achievable summary score was 14.

### Secondary endpoints

Secondary endpoint parameters were:

- Implant survival defined as time until occurrence of implant loss between T1 and T3.
- Volumetric soft-tissue changes at the implant site in the crestal (“upper”) and more apical (“lower”) region measured at T2 and T3.
- Patient satisfaction for the whole treatment reported at T3 using a numeric rating scale (NRS 0–10).
- Pain during the treatment using a numeric rating scale (NRS 0–10) reported at T3.
- Linear measurements in the CBCT at T1 and T3.

### Volumetric measurements

**Fig. 1:**
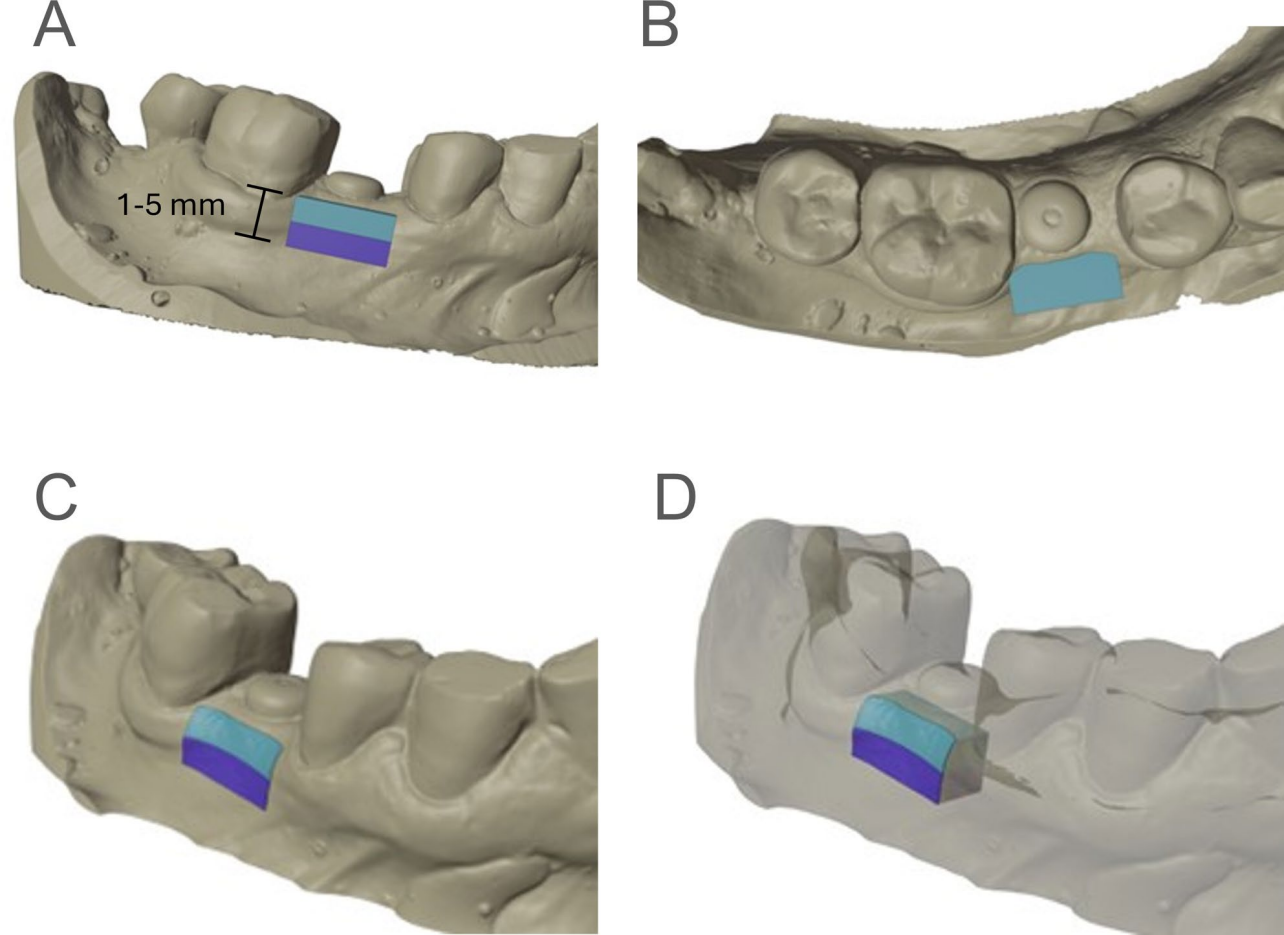
Workflow for the volume Measurement. The two standardized Volumes each 2 mm × 6 mm and extending from 1 mm apically in respect to the implant shoulder to 3 mm (light blue) and 3 mm apically to 5 mm (dark blue). **A** buccal view. **B** occlusal view. **C** After calculation of the Boolean intersection the distinct region of interest is visible. **D** Transparent view of the model with the intersection volumes opaque shows the extension of the region of interest extending to the alveolar ridge midline

Volumetric assessments were performed using digitized plaster models derived from impressions taken at time points T1, T2, and T3. The digitized plaster models were aligned to the T1 scan using the surrounding anatomy not affected by surgery. An initial, rough alignment was performed using a three-point alignment. Subsequently, further refinement was achieved by using an iterative closest point algorithm on the surrounding anatomy. The alignment was verified visually as well as by an average alignment error (mean squared error) of under 0.001. To allow for region-specific analysis of tissue dynamics, the volumetric assessment was divided into two distinct regions. This approach was chosen because the crestal region—located immediately apical to the implant shoulder—is particularly critical for esthetic outcomes, as it directly influences soft tissue support, papilla formation, and mucosal contour. The first standardized target volume was defined as 2 mm in height and 6 mm in width, aligned parallel to the occlusal plane, and positioned 1 mm apical to the implant shoulder and centered mesio-distally regarding the implant. A second measurement volume was established, also 2 mm in height, corresponding to a region from 3 mm to 5 mm below the implant shoulder. The depth of these volumes extended to the midline of the alveolar ridge referenced by the central fissure of the neighboring teeth. The plane limiting the bucco-lingual extension was constructed perpendicular to the occlusal plane. Volume determination was executed through Boolean operations on the digital models, enabling the calculation of volumetric differences between T1 and T2, as well as between T2 and T3. This methodology allowed for precise quantification of tissue changes over time (Fig. 1).

### Sample size calculation

For this exploratory study, a total of 25 patients per group were to be included. The sample size was based solely on feasibility considerations. With this number of patients, and assuming that only one implant per patient was examined, a difference of Cohen’s d = 0.8 between the groups for the primary endpoint “soft tissue stability during implant therapy” could be detected with a (descriptive) significance level of α = 5% and a power of 1-β = 79.1% using an unpaired two-sided Student’s t-test. Considering that the analysis of the primary endpoint was to be conducted using a multilevel model (to account for the possibility of including multiple teeth per patient), the study was expected to have an even higher power (assuming the same effect size of d = 0.8). These considerations were purely hypothetical due to the lack of prior empirical evidence upon which to base them, and merely illustrate the potential evidence that can be drawn from the study. As this was the first study of its kind, the final treatment effect size was still unknown. This study was intended to serve as a basis for further, larger confirmatory studies. Power calculation was performed using SAS version 9.4 (SAS Institute, Cary, NC, USA).

### Randomization

In total 48 implants were randomly allocated to one of the two study groups (open healing vs. closed healing) in a 1:1 ratio using block randomization with variable block lengths of 4, 4 and 2. To maintain allocation concealment, the block size was not disclosed to study personnel. A randomization list was generated by the Institute of Medical Biometry, and sequentially numbered, opaque, sealed envelopes were prepared accordingly. Once a patient met the inclusion criteria and was enrolled in the study, their name was added to a centralized study list. On the day of surgery, the attending clinician opened the next envelope in the predefined sequence to reveal the treatment allocation.

### Drop-outs

At baseline, two patients dropped out of the open and closed healing group, respectively. In the open healing group, one patient aborted the treatment whereas the other did not show up. In the closed healing group, both patients aborted the treatment. At the first follow-up (T2) one patient did not want to continue treatment in the closed healing group. At the second follow-up examination (T3), one patient died in the open healing group for unrelated reasons. Hence, the total number of available implants was as follows:

- Open healing group: 25 implants at T1, 23 at T2, and 21 at T3.
- Closed healing group: 23 implants at T1, 21 at T2, and 20 at T3.

**Fig. 2:**
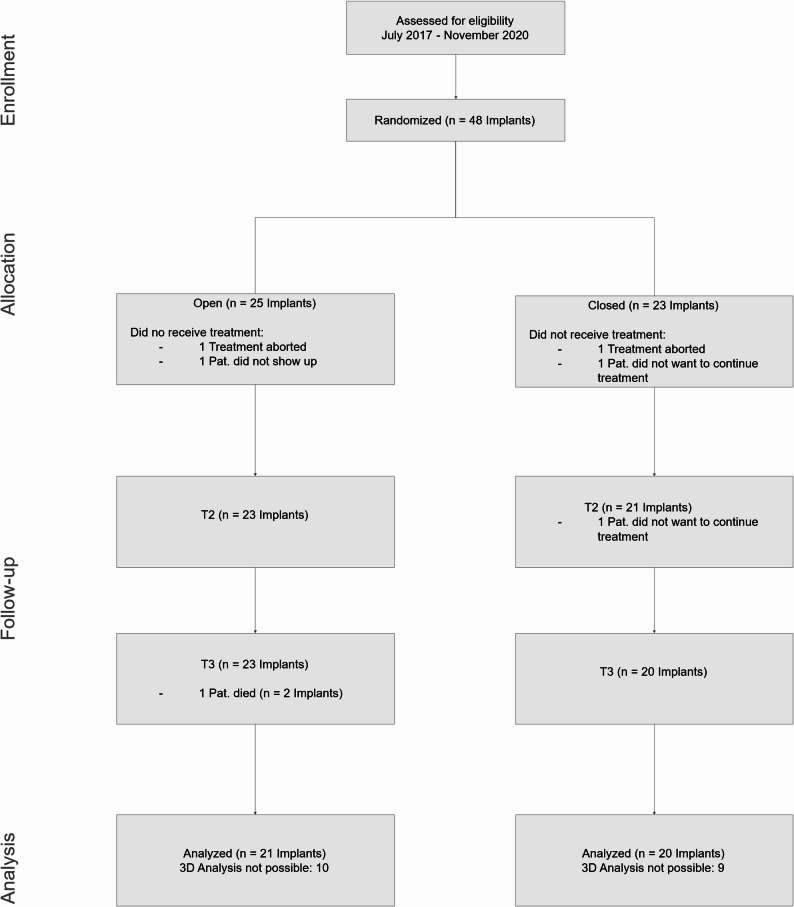
Participant flow diagram of the randomized controlled trial. In total 48 Implants were randomized into a study and a control arm. Patients were followed up for one year for 41 implants in total

In total, 41 of the initially randomized 48 implants (85.4%) were followed through the complete study period and included in the per-protocol analysis. A detailed overview of participant flow and implant availability at each time point is presented in Fig. 2.

### Surgical procedure and follow-up

Under local anesthesia, the tooth was extracted, minimizing trauma to the surrounding hard and soft tissue to ensure complete preservation of the alveolar bone. Immediately following extraction, the socket was thoroughly debrided of inflammatory tissue using a sharp curette, and the buccal wall was probed to confirm bone integrity.

Implant osteotomy was initiated with a palatal orientation of the implant axis. Once the desired length and diameter were achieved, the implant (K3, Argon Dental Vertriebs GmbH, Bingen am Rhein, Germany) was inserted. The buccal gap between the implant shoulder and the buccal bone was subsequently filled with 90% demineralized bovine bone and 10% bovine collagen (BioOss Collagen, Geistlich Biomaterials, Wolhusen, Switzerland).

In the “closed healing“ group, a full-thickness mucoperiosteal flap was elevated using vertical incisions, followed by a periosteal releasing incision to allow tension-free repositioning. After implant placement and the insertion of a cover screw, the flap was repositioned over the implant, enabling submerged healing. No cases of wound dehiscence were observed in this group during the healing period.

In the “open healing“ group, a flapless approach was employed. In this group, the implant was sealed with a healing abutment, and the soft tissue was adapted around the abutment using adaptive suturing techniques, thereby obviating the need for flap elevation. Single interrupted sutures were used for all suturing purposes using Vicryl 4 − 0 (Ethicon, Johnson & Johnson Medtech, Somerville, NJ, USA). After 3 months, the healing abutment was inserted in the closed healing group using a small crestal incision and minimal soft tissue mobilization. Using single interrupted sutures, the gingiva was adapted to the abutment.

The healing abutments were chosen to match the diameter of the implant and the gingival thickness, ensuring that one millimeter of the healing abutment protruded vertically.

### Statistical methods

Continuous variables are expressed as mean ± standard deviation (SD) or median (Q1–Q3), and categorical variables as absolute and relative frequencies. Appropriate testing of distribution of continuous variables and corresponding tests (Student’s t-test or Mann-Whitney-U-test) were performed. For categorical variables, Pearson’s χ2 test was used. In addition, the number of missing values was added (if present).

The primary endpoint, PES, was analyzed using a linear mixed effect model. Using the PES summary from visits T2 and T3 as the dependent variable, the following independent variables (fixed effects) were used: Group (open/closed healing) as well as adjusting variables PES summary at baseline (T1), timepoint of PES measurement (T2/T3), Smoking (yes/no), mesial height at T1 and sex (male/female). As multiple measurements per participant were recorded, a random intercept effect was added for the participants to take the heteroskedasticity of all participants into account. The model was fitted using restricted maximum likelihood (REML) and p-values and 95% profile likelihood confidence intervals were calculated. Furthermore, the assumptions of the linear regression (linearity, influential observations, homogeneity of variance, collinearity, normality of residuals) were verified.

Moreover, using the sample size and the observed Cohen’s d effect measure of the treatment group regarding PES summary, a post-hoc power analysis for an unpaired two-sided Student’s t-test was performed. The observed Cohen’s d of the treatment group regarding PES summary was computed by dividing the estimated difference of marginal PES summary mean between the treatment groups (open vs. closed healing) by the residual standard deviation of the previously described linear mixed model, yielding a within-subject standardized effect size.

As this was an explorative study, the p-values obtained were interpreted purely descriptively, and alpha adjustment for multiple testing was not performed. A p-value smaller than 0.05 was considered statistically significant. Statistical analyses were conducted using the statistical software R (version 4.2.2, R Core Team, Auckland, New Zealand) using the packages “lme4” and “lmerTest” for linear mixed effect models, “performance” to validate the regression assumptions as well as “ggplot” for data illustrations.

## Results

The study included 46 patients and 48 implants, divided into two groups: open healing (*n* = 25) and closed healing (*n* = 23). Both groups demonstrated comparable distributions in implant site locations, baseline health conditions, and habits such as smoking. However, there was a higher percentage of male participants in the closed healing group (83%) compared to the open healing group (56%) (see Table 1). No medically significant baseline differences were noted in peri-implant soft tissue or bone parameters, ensuring comparability between groups. All available data were analyzed (Fig. 2).

**Table 1:** Description of study population divided into treatment groups. SD: standard deviation. 1Wilcoxon rank sum test

	Open (*n* = 25)	Closed (*n* = 23)	Total (*n* = 48)
*Tooth*			
12	0	1 (4%)	1 (2%)
14	0	2 (8%)	2 (4%)
15	10 (40%)	3 (12%)	13 (27%)
21	1 (4%)	0	1 (2%)
22	3 (12%)	0	3 (6%)
23	1 (4%)	0	1 (2%)
24	3 (12%)	1 (4%)	4 (8%)
25	2 (8%)	6 (26%)	8 (17%)
35	3 (12%)	5 (22%)	8 (17%)
44	0	1 (4%)	1 (2%)
45	2 (8%)	4 (17%)	6 (12%)
Active smoker	18 (72%)	18 (78%)	36 (75%)
*Sex*			
M	14 (56%)	19 (83%)	33 (69%)
F	11 (44%)	4 (17%)	15 (31%)
*Implant diameter*			*p*-value^1^ = 0.2758
3.5 mm	1 (4%)	1 (4.35%)	2 (4.17%)
4 mm	7 (28%)	10 (43.48%)	17 (35.42%)
4.5 mm	10 (40%)	8 (34.78%)	18 (37.5%)
5 mm	6 (24%)	3 (13.04%)	9 (18.75%)
5.5 mm	1 (4%)	1 (4.35%)	2 (4.17%)
Mean ± SD	4.48 ± 0.47	4.35 ± 0.46	4.42 ± 0.47
*Implant length*			
8.5 mm	1 (4.00%)	0 (0.00%)	1 (2.08%)
9 mm	0 (0.00%)	1 (4.35%)	1 (2.08%)
10 mm	3 (12.00%)	4 (17.39%)	7 (14.58%)
11.5 mm	5 (20.00%)	3 (13.04%)	8 (16.67%)
13 mm	10 (40.00%)	10 (43.48%)	20 (41.67%)
15 mm	6 (24.00%)	5 (21.74%)	11 (22.92%)
Mean ± SD	12.64 ± 1.82	12.54 ± 1.83	12.59 ± 1.81

### Primary endpoint: pink esthetic score (PES)

All assumptions of the linear regression (linearity, influential observations, homogeneity of variance, collinearity, normality of residuals) were fulfilled. The initial PES at T1 was 9.2 ± 2.5 in the open healing group and 8.6 ± 2.7 in the closed healing group.

The linear mixed-effects model revealed a statistically significant difference in PES outcomes between the open and closed healing techniques. The open healing group showed a superior PES summary compared to the closed healing group, with an adjusted estimate of -1.4863 (95% CI: [-2.3564; -0.6161], *p* = 0.0014) favoring open healing. Assuming a sample size of 41 patients per group and an observed Cohen’s d of the treatment group regarding PES summary of 0.78, the post-hoc power analysis yielded a 93.5% power to detect treatment group difference (open vs. closed healing) in PES summary when conducting an unpaired two-sided Student’s t-test at the 5% significance level. Additionally, the linear mixed-effects model showed that the PES was significantly dependent on the PES at T1 (0.3638, 95% CI: [0.1890; 0.5386], *p* = 0.0002).

Smoking status (-0.1726, 95% CI: [-1.1241; 0.7789], *p* = 0.7142) and mesial tissue height (0.0011, 95% CI: [-0.0560; 0.0582], *p* = 0.9683) (Appendix Table A1) at baseline and sex (0.8439, 95%-CI: [-0.0716; 1.7594], *p* = 0.0696) did not significantly influence PES outcomes (Table 2).

**Table 2:** Linear mixed effect model for PES summary (*n* = 82). Estimated variance of random intercept: 0.3413. Legend: *Significance value of < 0.05; **Significance value of < 0.001; PES: Pink esthetic score; CL: confidence level

	Estimate	Lower CL	Upper CL	*p*-Value
Intercept	3.6964	1.7487	5.6441	0.0004**
*Group*				
Open Healing	-	-	-	-
Closed Healing	-1.4863	-2.3564	-0.6161	0.0014*
PES summary score T1	0.3638	0.1890	0.5386	0.0002**
*Time point*				
T2	-	-	-	-
T3	2.8637	2.1454	3.5820	< 0.0001**
*Smoking*				
Yes	-	-	-	-
No	-0.1726	-1.1241	0.7789	0.7142
Mesial height T1	0.0011	-0.0560	0.0582	0.9683
*Sex*				
Female	-	-	-	-
Male	0.8439	-0.0716	1.7594	0.0696

### Secondary endpoints

The implant survival rate was 100% in both groups, with no implant losses reported during the study period. Differences in soft tissue parameters, including mesial and distal papilla levels, were more pronounced in the open healing group, showing higher frequencies of “complete” papillae at T3 (79% vs. 65% for mesial papilla; 21% vs. 5% for distal papilla). However, distal papilla outcomes remained challenging in both groups, with higher rates of “missing” scores persisting over time (23% at T3 overall).

The height, color, and texture of peri-implant soft tissues were evaluated over time:

Contour: A “natural” soft tissue contour was more frequently observed in the open healing group at T3 (68% vs. 30%), with the closed healing group showing a higher prevalence of “unnatural” contours (10% vs. 0%).

**Fig. 3:**
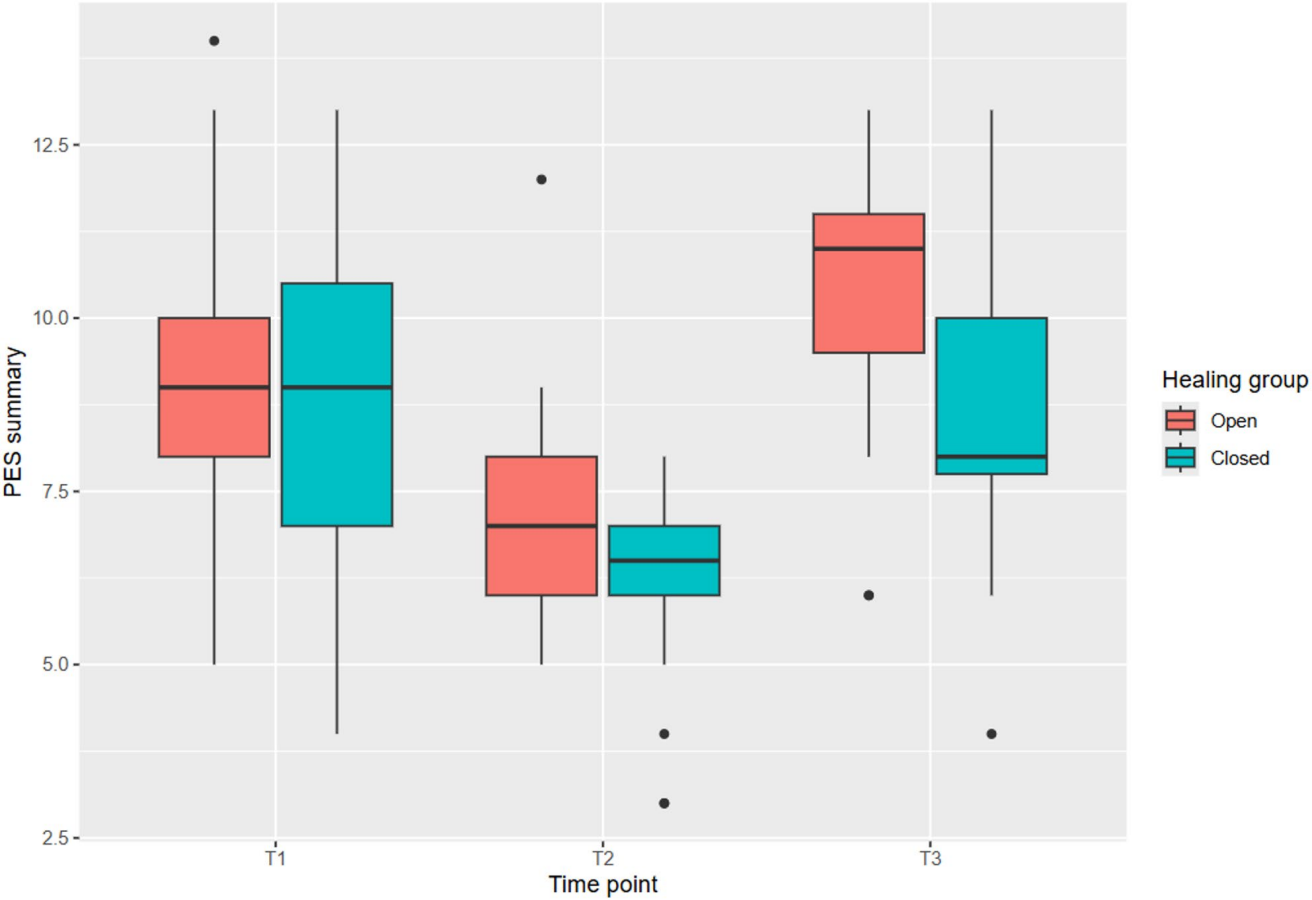
Comparison of the two study groups and the Pink Esthetic Score (PES) at the three different time points (T1–T3). Starting with comparable PES distributions, the open healing group achieved statistically significantly higher PES values at the one-year follow-up (T3)

Color and Texture: Good tissue color was observed in 47% (open) and 35% (closed) at T3, while good texture was reported in 68% (open) and 40% (closed), favoring open healing (Fig. 3).

### Volumetric analysis

Volumetric changes were measured at three time points (T1, T2, and T3) to assess tissue stability.

**Fig. 4:**
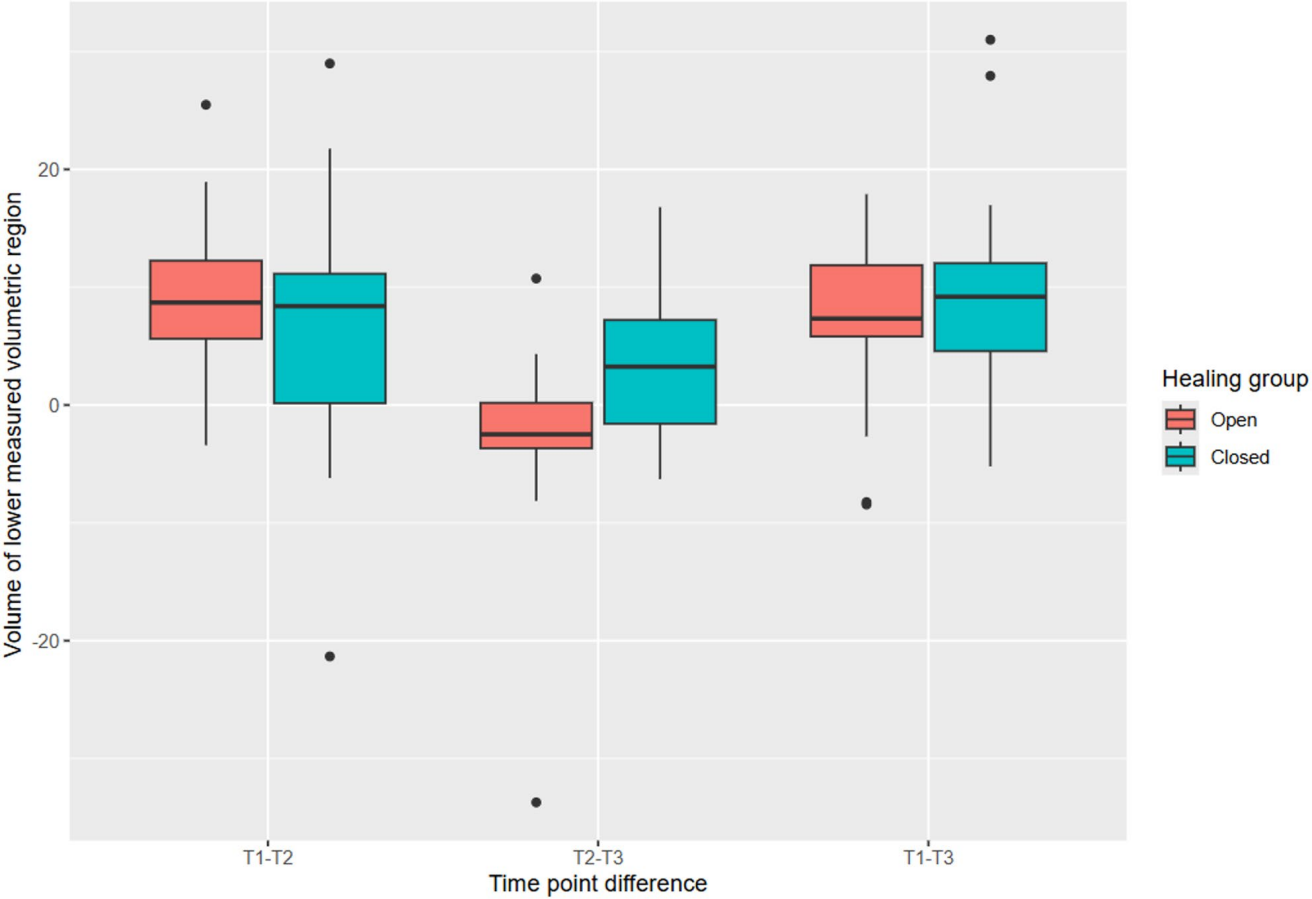
Development of the volume of the lower measured volumetric region (3–5 mm apically from implant shoulder). Between T1 and T2 the average volumetric loss was 14 mm³ ± 7.6. Between T2 and T3 the volumetric changes were smaller at -4.3 mm³ ± 9.2

Volumetric loss (apical region): from T1 to T2, no significant difference was found between groups (open: 9.5 mm³ ± 6.3, closed 6.4 mm³ ± 12, *p* = 0.323). However, from T2 to T3, the closed healing group showed a statistically significant improvement compared to the open healing group (open: -3.3 mm³ ± 10, closed: 4.1 mm³ ± 7.2, *p* = 0.025). Overall differences from T1 to T3 were not statistically significant (open: 6.4 mm³ ± 8.1, closed: 10 mm³ ± 10, *p* = 0.579) (Fig. 4).

**Fig. 5:**
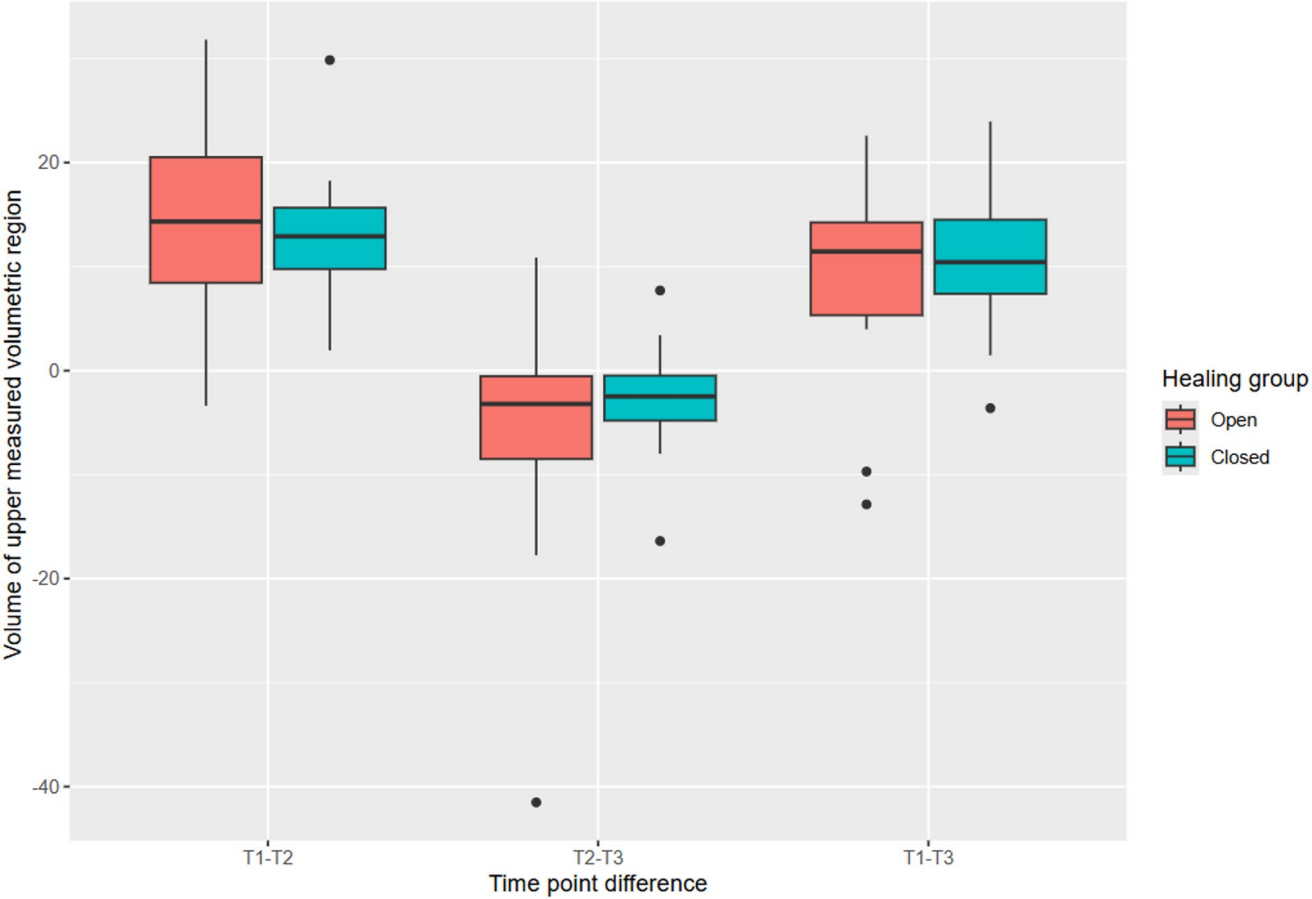
Development of the volume of the upper measured volumetric region (1–3 mm apically from implant shoulder). Between T1 and T2 the average volumetric loss is 8.2 mm³ ± 9.2. Between T2 and T3 the volumetric changes reduce and the difference is at 0.58 mm³ ± 9.5

Volumetric loss (crestal region): the open healing group demonstrated slightly better stability from T1 to T3, with mean differences of 8.8 mm³ ± 9.5 (open) vs. 10 mm³ ± 7.2 (closed), though these differences were not statistically significant (*p* > 0.999) (Table 3; Fig. 5).

**Table 3:** Measurements of the volumes (upper and lower buccal implant region). Upper volume: 1–3 mm apically from implant shoulder; lower volume: 3–5 mm apically from implant shoulder; MWU: Mann-Whitney’s U Test; * = p-value < 0.05

Variable	Open	Closed	Total	*p*-value (MWU)
Difference T1–T2 upper volume (mm³)	14 ± 8.4 (*n* = 19)	13 ± 6.8 (*n* = 15)	14 ± 7.6 (*n* = 34)	0.732
Difference T2–T3 upper volume (mm³)	-5.8 ± 12 (*n* = 15)	-2.7 ± 5.4 (*n* = 15)	-4.3 ± 9.2 (*n* = 30)	0.412
Difference T1–T3 upper volume (mm³)	8.8 ± 9.5 (*n* = 15)	10 ± 7.2 (*n* = 15)	9.5 ± 8.3 (*n* = 30)	> 0.999
Difference T1–T2 lower volume (mm³)	9.5 ± 6.3 (*n* = 19)	6.4 ± 12 (*n* = 13)	8.2 ± 9.2 (*n* = 32)	0.323
Difference T2–T3 lower volume (mm³)	-3.3 ± 10 (*n* = 13)	4.1 ± 7.2 (*n* = 14)	0.58 ± 9.5 (*n* = 27)	0.025*
Difference T1–T3 lower volume (mm³)	6.4 ± 8.1 (*n* = 13)	10 ± 10 (*n* = 13)	8.4 ± 9.3 (*n* = 26)	0.579

### Patient reported outcomes

Pain scores at T3 were generally low in both groups, with no significant differences. Patient-reported esthetic satisfaction at T3 showed high scores in both groups, with 74% in the open healing group and 60% in the closed healing group rating their satisfaction as “10” on a 10-point scale (Table 4). Apart from loss to follow-up, two patients in the open healing group did not complete the PROM questionnaire, leading to missing values.

**Table 4:** Reports of the pain and esthetic outcome by patients using the NRS (0–10). Pearson’s Chi-squared test

Characteristic	Open (*n* = 25)	Closed (*n* = 23)	Total (*n* = 48)	*p*-value^1^
*Pain*				0.1100
0	6 (31.58%)	3 (15.00%)	9 (23.08%)	
1	5 (26.32%)	5 (25.00%)	10 (25.64%)	
2	3 (15.79%)	10 (50.00%)	13 (33.33%)	
>= 3	5 (26.32%)	2 (10.00%)	7 (17.95%)	
Missing	6	3	9	
*Esthetic*				0.5379
8	1 (5.26%)	3 (15.00%)	4 (10.26%)	
9	4 (21.05%)	5 (25.00%)	9 (23.08%)	
10	14 (73.68%)	12 (60.00%)	26 (66.67%)	
Missing	6	3	9	

## Discussion

The results of this study offer valuable insights into the comparative effectiveness of open and closed healing techniques in IIP. The primary finding, that the open healing technique results in superior esthetic outcomes as measured by the PES, is significant for clinical decision-making, particularly for patients who prioritize esthetics. Statistical analysis showed a statistically significant increased PES summary for the open compared to the closed techniques (-1.4863, 95% CI: [-2.3564; -0.6161], *p* = 0.0014).

A post hoc power analysis based on the observed Cohen’s d of 0.78 and the available sample size indicated high statistical power to detect a between-group difference in PES. The magnitude of the observed effect may serve as a reference for sample size planning in future confirmatory trials. However, given the exploratory nature of the study and the known tendency for effect sizes to be overestimated in initial investigations, future randomized controlled trials should adopt more conservative effect size assumptions when calculating sample size.

The finding that a better initial PES predicts a better outcome (2.8637, CI: [0.1890; 0.5386], *p* = 0.0002) suggests that early attention to soft tissue condition can have an impact on esthetic outcomes which persist over the long-term. This underscores the importance of preoperative evaluation and planning, as initial soft tissue health influences overall success with regard to healing and implant integration. This can also be seen at the level of the poor scores for the distal papillae which persisted throughout the study and across the groups. A well-established option in patients with challenging soft-tissue esthetics is the option of connective tissue grafting, which was outside the scope of this study.

In terms of volumetric stability, both healing techniques experienced a period of volume loss between T1 (pre-extraction) and T2 (3 months post-operation), which then stabilized or slightly improved by T3 (12 months post-operation). This is a common finding, as tissue tends to undergo remodeling in the early phases of healing, but the trend of volume recovery seen here is promising, particularly in the context of long-term implant success [13]. The open healing technique demonstrated slightly less volume loss (8.8 mm³ ± 9.5 vs. 10.0 mm³ ± 7.2), although this difference was not statistically significant. As shown in Fig. 2, however, a subset of cases had to be excluded from the 3D volumetric analysis due to technical limitations. Specifically, 10 cases in the open healing group and 9 cases in the closed healing group could not be analyzed due to either insufficient extension of the plaster models in the apical region or other impression artefacts. These limitations prevented reliable alignment and volumetric measurement in the affected cases. While the distribution of missing data was similar between groups, minimizing the risk of systematic bias, the reduced sample size may limit the statistical power of volumetric analysis and should be considered when interpreting these results.

The clinical relevance of this finding suggests that both techniques are effective at preserving tissue volume over time, even if the open technique offers a slight advantage. The comparison of open and closed healing techniques includes the effects of flap elevation for the closed healing group. Our results are in alignment with the research and hypothesis that flap elevation results in increased buccal shell resorption and therefore jeopardizes the esthetic result.

The literature identifies several additional factors that can positively impact the esthetic outcome of IIP. For instance, grafting the buccal gap has been shown to limit resorption and enhance volumetric stability–even though smaller gaps may heal without grafting [14, 15]. Furthermore using a socket shield technique to stabilize the buccal alveolar bone has been discussed in the literature [16]. In this study, both groups were treated following identical grafting protocols to control for this variable. Similarly, while the use of a connective tissue graft can increase buccal tissue volume, particularly in patients with a thin phenotype prone to pronounced resorption, no soft-tissue grafting was performed here, thereby eliminating this factor as a potential confounder [17, 18].

Regarding sex as a potential confounder, the male-to-female ratio differed between the groups. It approached statistical significance (*p* = 0.0696) between the two groups. Given that soft-tissue biotype is known to differ between sexes—with males generally exhibiting thicker tissue phenotypes—this imbalance could have influenced the esthetic outcomes. However, because sex was included as an independent variable in the mixed-effects model, its potential confounding effect was statistically adjusted for in the analysis.

This study was registered retrospectively because, at the time of its conception, trial registration was not routinely required for exploratory single-center, investigator-initiated studies in implant dentistry at our institution. Nonetheless, the study followed a prospective, randomized design with prior ethical approval. Retrospective registration (DRKS00038390) was completed to align with current standards for research transparency.

Provisionalization has been shown to promote tissue preservation and enhance esthetic outcomes, however, this approach necessitates sufficient insertion torque [16, 19, 20]. When insertion torque is insufficient, alternative workflows must be considered, for example an individualized healing abutment can also be considered [2]. The workflow presented herein deliberately excluded provisionalization to evaluate non-provisionalized IIP.

Finally, implant positioning is a critical determinant of both esthetic outcomes and the remodeling of hard and soft tissues. Implants placed too buccally or with an excessively large diameter may predispose to increased resorption and subsequent gingival recession [21]. Notably, implants with a buccal shoulder position have been associated with a significantly higher incidence of facial mucosal recession. Evans and Chen reported recession in 80% of IIPs with buccal positioning, compared to 28.13% when optimal positioning was achieved [22].

In addition, papilla fill also depends on the clinical attachment levels of the neighboring teeth and the distance between the marginal bone peak and the contact point of the clinical crown [21]. A vertical distance of more than 5 mm can lead to incomplete papilla filling, which was evident in the PES evaluation [23]. Notably, 72% of the open healing group and 78% of the closed healing group were smokers, although strong smokers (> 10 cigarettes/day) were excluded from the study. While this smoking rate is higher than in the general population, smoking status was included in the statistical model and did not significantly affect esthetic outcomes. Nonetheless this may affect the esthetic results, i.e. the papilla fill.

The absence of implant losses in both groups is noteworthy, demonstrating the viability and high survival rate of immediate implants when the technique is carefully selected for suitable patients. This high survival rate across both healing techniques indicates that IIP can be reliable for a range of patients, given adequate peri-implant tissue stability [24].

Overall, the findings of this study contribute to the ongoing debate about the optimal healing technique for immediate implants and provide guidance for clinical decision making. While both methods appear effective in terms of implant survival, the esthetic advantages of the open healing technique, given the small differences in volumetric changes, may make it the preferred choice for patients and clinicians aiming for superior esthetic outcomes. These improved outcomes may be attributed to avoiding a flap elevation procedure and, therefore, periosteal elevation of the buccal implant wall, which may preserve buccal bone vascularization and therefore reduce the rate of bone resorption.

## Conclusions

Further research with larger sample sizes and longer follow-up periods could help to confirm these findings and potentially explore other factors, such as patient satisfaction and cost-effectiveness. The study revealed that the open healing technique offers superior esthetic outcomes compared to the closed healing technique in IIP. 

## Data Availability

The datasets used and analyzed in the presented study are available from the corresponding author on reasonable request.

## Appendix

See Table 5.

**Table 5:** Table of bone height measured in CBCT scans at T1 and T3

Mesial height	*n* = 23	*n* = 21	*n* = 44
T1	16 mm ± 7.3	18 mm ± 7.5	17 mm ± 7.4
T3	15 mm ± 4.7	20 mm ± 8.2	17 m ± 7.1
**Distal height**	*n* = 21	*n* = 20	*n* = 41
T1	16 mm ± 7.3	18 mm ± 7.5	17 mm ± 7.4
T3	14 mm ± 6.2	18 mm ± 7.6	16 mm ± 7.1

## Abbreviations

CBCT: Cone beam computed tomography
CONSORT: Consolidated Standards of Reporting Trials
FDI: Fédération dentaire internationale
IIP: Immediate implant placement
NRS: Numeric rating scale
PES: Pink Esthetic Score
STL: Standard Triangle Language

## Declarations

Institutional Review Board Statement: The study was conducted in accordance with the Declaration of Helsinki, and approved by the Ethics Committee of the Medical Faculty of the University of Heidelberg (application S-230/2017).
